# Dysembryoplastic neuroepithelial tumor and probable sudden unexplained death in epilepsy: a case report

**DOI:** 10.1186/1752-1947-5-441

**Published:** 2011-09-07

**Authors:** Carmen-Adella Sîrbu

**Affiliations:** 1 Central Military Emergency University Hospital, "Dr Carol Davila" Department of Neurology, Calea Plevnei 134, Bucharest, Romania

## Abstract

**Introduction:**

This is the first report of the case of a patient with a natural history of dysembryoplastic neuroepithelial tumor associated with probable sudden unexplained death in epilepsy. These tumors are benign, arising within the supratentorial cortex. Over 100 cases have been reported in the literature since the first description by Daumas-Duport in 1988.

**Case presentation:**

A 24- year-old Caucasian woman had a long period of intractable complex partial seizures, sometimes with tonic-clonic generalization and neuropsychological abnormalities. Magnetic resonance imaging showed a cortico-subcortical parietal tumor with all the characteristics of these types of tumors. After 14 years of evolution, our patient died suddenly during sleep.

**Conclusion:**

To the best of our knowledge, this is the first case of probable sudden unexplained death in symptomatic epilepsy due to dysembryoplastic neuroepithelial tumor with natural history. Early and complete excision, with functional studies before and during the surgery, leads to better control of seizures, avoiding neuropsychological changes and the risk of death. Patients with refractory epilepsy should be evaluated for any sleep disorders and should have complete cardiology assessments including electrocardiographic evaluation of cardiac rhythm disturbances.

## Introduction

Dysembryoplastic neuroepithelial tumor (DNT) is a rare low-grade, mixed neuronal and glial tumor, usually associated with pharmacologically intractable, complex partial or generalized seizures which date from childhood. DNTs are heterogenous lesions composed of multiple, mature cell types. Features include a multinodular and multicystic appearance, the presence of both neuronal and glial (oligodendrocytic and astrocytic) components with little if any cytologic atypia, the presence of accompanying cortical dysplasia, and the lack of an arcuate vascular pattern. The prognosis after surgery is favourable. Differentiation of DNT from gangliogliomas or other low grade gliomas is possible using magnetic resonance imaging (MRI) features and is important because DNT does not recur after epilepsy surgery. Therefore postoperative radiation and chemotherapy are not needed, and in infancy and childhood they may be deleterious, so the recognition of surgically curable clinicopathological entities is mandatory. The presence of secondary generalized seizures, an extratemporal irritative zone and a structural lesion in extratemporal regions correlate with sudden unexplained death in epilepsy (SUDEP). Thus, all efforts should be undertaken to eliminate this seizure including abstract epilepsy surgery.

## Case presentation

**Figure 1:**
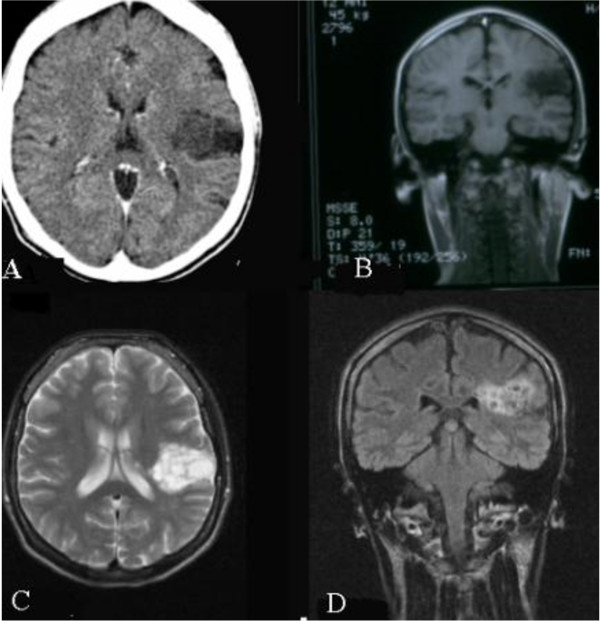
**Imaging results**. (A) First CT scan show a left temporoparietal diffuse hypodense area, quite inhomogeneous without mass effect. (B- D) MRI performed 13 years after seizure onset revealed a multicystic cortico-subcortical parietal lesion, without edema, mass effect, and enhancement.

A 24- year-old Caucasian woman was admitted to our department with refractory epilepsy. Her history included a normal birth and normal psychomotor development. The seizures started at the age of 11, and were of the complex partial atonic type. The moment of mental decline and change of behavior appeared a few months after the onset of seizures. Computer tomography (CT) showed a left temporoparietal diffuse hypodense area, quite inhomogeneous without mass effect (Figure 1, panel A). Cerebral MRI performed four years later confirmed the diagnosis of brain tumor. Over this time, the pattern of the seizures changed, becoming partial, complex, sometimes evolving to secondarily generalized seizures, with premenstrual flare-ups in intensity and frequency. Surgery or brain biopsy were constantly refused by the patient's mother. She was treated with carbamazepine, phenytoin, valproic acid and topiramate in diverse doses and combinations without effect on seizures, which continued once or several times a day.

On admission to our clinic, 13 years after the disease onset, neurological examination revealed no positive findings other than neuropsychological abnormalities. Mnesic activity, general cognitive index (GCI), vocabulary and operational effectiveness of thinking had decreased by 35% (mean range) compared to the previous examination at disease onset.

**Figure 2:**
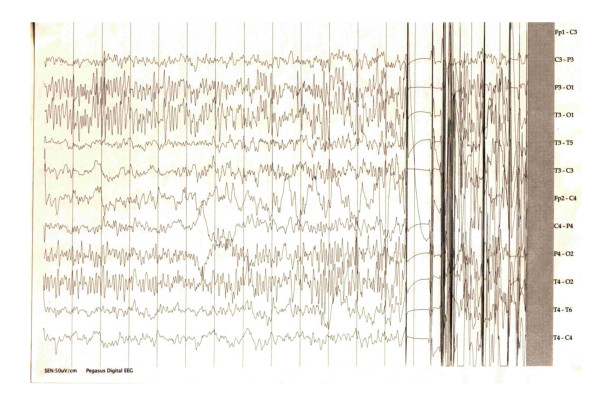
**EEG showing interictal spikes and polyspikes**. One minute of hyperventilation activated a tonic-clonic generalized seizure.

Biological tests appeared to be normal. A chest X-ray and cardiology examination were normal. MRI revealed a 32.3 mm (anteroposterior)×43.1 mm (transverse)×28.3 mm (craniocaudal) multicystic cortico-subcortical parietal lesion, divided by septations, without edema or mass effect, and no enhancement (Figure 1, panels B, C, D). As our patient refused to have a cerebral biopsy, we decided to perform a complementary imaging exploration, which could offer us more details about the tumor. Tomoscintigraphy (single-photon emission CT) with Tc99m MIBI indicated no tumor metabolic activity. Standard electroencephalogram (EEG) showed interictal abnormalities like spikes and polyspikes. One minute of hyperventilation activated a tonic-clonic generalized seizure, accompanied by specific EEG recording (Figure 2). The long history together with the clinical and imaging data led us to the diagnosis of DNP. One year later, our patient died during sleep. At that time she was on topiramate 400 mg/day in two divided doses, without seizure control.

## Discussion

To the best of our knowledge, this is the first reported case with probable sudden death in symptomatic epilepsy due to DNT. DNT is a newly-described, pathologically benign tumor, arising within the supratentorial cortex. Over 100 cases have been reported in the literature since the first description by Daumas-Duport in 1988 [1]. The majority of cases are found in the temporal lobe where they can coexist with mesial temporal sclerosis, followed by the frontal, parietal and rarely the occipital lobe. From the epidemiologic point of view, incidence is between six and 35 years old, with an average of 21.5 years and an equal sex distribution.

DNT has a multinodular architecture, mainly in the cortex, and consists of oligodendrocytes, astrocytes, neurons, and glyconeural elements. This cortical structural abnormality disrupts normal neuronal circuitry and becomes an epileptogenic focus. Neuronal cells in the lesion may also secrete neurotransmitters or express receptors. Neuronal markers (synaptophysin, neuron- specific enolase) and glial markers (GFAP, S-100) are positive. Despite benign behavior, it may have a high MIB-1 labeling index. Aberrant expression of apoptosis-associated proteins (bcl-2, bcl-x, bax), similar to what has been previously described in gangliogliomas (another epilepsy-related, dysplasia-associated tumor), may play a role in the pathogenesis of DNT [2]. Today, DNT refers to polymorphic tumors that appear during embryogenesis. In the revised World Health Organization classification, DNTs have been incorporated into the category of neuronal and mixed neuronoglial tumors [3]. Non contrast-enhanced CT scans show well-demarcated lesions that are hypodense relative to the surrounding brain, sometimes with intratumoral calcification and multicystic appearance. Routine MRI sequences reveal a well-demarcated lesion, hypointense on T1-weighted images, and hyperintense on T2-weighted images. Edema and mass effect on midline structures are lacking, although they may be observed in cases of hemorrhagic complications [4]. The lobular aspect with presence of septations can sometimes occur (as in our case). Single-photon emission CT has been used in limited fashion with DNTs, and this shows hypoperfusion or poor isotope uptake. MR spectroscopy allows the determination of certain biochemical properties of the brain *in vivo *and reflects the biologic characteristics of benign tumor. The combination of preoperative positron emission tomographic metabolic studies with functional brain mapping allows for prediction of tumor type, defines eloquent areas of cortical function, and improves approach and resection of the tumors with minimal risk of neurological impairment. Differential diagnosis includes oligodendrogliomas, mixed gliomas and gangliogliomas. Treatment options and prognosis differ significantly between these lesions. It is important that DNT and glioma be correctly differentiated at diagnosis, because patients with DNT should not be subjected to potentially harmful adjuvant therapies such as radiation or chemotherapy.

Our diagnosis was based on the characteristic imaging investigations, the stationary dimensions of the tumor during a follow-up of 13 years and the clinical expression of epilepsy unresponsive to treatment. It is true that a morphopathological examination would have helped to confirm the diagnosis, although this may sometimes be irrelevant.

Treatment for DNT is surgical resection; however, there is no cohort of untreated control patients. The relationship of DNT to the epileptogenic foci can be determined by extensive interictal and ictal EEG recordings. Noninvasive recording and careful mapping show that a structural lesion is not the source of epileptic activity. There is little correlation between the lesion site and epileptogenic foci of the ictal onset zone as well as the irritative zone. Seizure control after surgery is good with 80-90% seizure free. Other authors show that seizure outcome is not always favorable. Although the majority of children remain seizure free after surgical excision of DNTs, a considerable number have recurrent seizures. Short-term outcome is influenced by older age at surgery and longer duration of epilepsy. Residual tumor is a significant risk factor for poor seizure outcome [5]. DNTs have a benign course, but there are some reports with malignant transformation. Our patient was found by her mother in a prone position at the time of death. At the time she was on topiramate 400 mg/day in two divided doses, without seizure control. Our patient meets the criteria used in most SUDEP studies: she had recurrent unprovoked seizures, died unexpectedly and suddenly while in a reasonable state of health, during normal and benign circumstances, and the death was not the direct result of a seizure or status epilepticus. The probable SUDEP is given because of lack of autopsy. In 60% of cases, the event was related to sleep, which might indicate involvement of a sleep-related event. Our patient was not assessed for any sleep disorders which may predispose to SUDEP.

The seizures are known to cause central apnea by direct propagation of the electrical discharge to the respiratory center. Cardiac dysrhythmias during the interictal state is another potentially fatal condition because of chronic autonomic dysfunction, effects of antiepileptic medication and a common genetic susceptibility [6,7]. Asystole might underlie many of the deaths. Clinical characteristics of patients with periictal cardiac abnormalities are very similar to those at greatest risk of SUDEP. Cardiac arrest can cause secondary cardiopulmonary arrest [8]. Asphyxiation secondary to an obstructive cause has been postulated to play a role in the deaths of patients who were found in a prone position at the time of death [9]. Human and animal data suggest that specific genetic factors might play a role in some cases. Serotonin might affect respiratory mechanisms and may be involved [10]. Prolonged postictal generalized electroencephalographic suppression, greater than 50 seconds, appears to identify refractory epilepsy patients who are at risk of SUDEP [11]. There are some data suggesting that having an extratemporal focus or lesion is the main correlate of SUDEP [12].

Our patient presented several risk factors: generalized seizures, lower age of onset of seizures, duration of seizures longer than 10 years, age between 20 and 40 years and a poorly controlled disorder. SUDEP incidence rates vary from 0.35 per 1000 person-years of follow-up in population based studies to 9.3 per 1000 person-years in patients with refractory epilepsy [13]. Estimated SUDEP rates in patients receiving the new anticonvulsant drugs lamotrigine, gabapentin, topiramate, tiagabine, and zonisamide were found to be similar to those in patients receiving standard anticonvulsant drugs, suggesting that SUDEP rates reflect population rates and not a specific drug effect. The Food and Drug Administration require warning labels on the risk of SUDEP in association with the use of each of the above-mentioned drugs [14]. Elimination of seizures after surgery reduces mortality rates in individuals with epilepsy to a level indistinguishable from that of the general population [15]. The published National Institute for Clinical Excellence guidelines state that "individuals with epilepsy and their families and/or carers should be given and have access to information on SUDEP".

## Conclusion

DNTs are now known to be more frequent in children and young adults than was previously believed. Early and complete surgery, with functional studies before and during the surgery, leads to a good control of seizures, avoiding complications such as hemorrhage, malignant transformation and neuropsychological changes, as in our case. Unfortunately, all the studies, (especially the case series) published so far mention only the medium term seizure control but do not refer to the neurological disabilities caused by the surgery. Patients with refractory epilepsy should have complete sleep disorder and cardiology assessments including electrocardiogram evaluation of cardiac rhythm disturbances, which could be performed at the same time as the EEG.

## Consent

Written informed consent for publication from the patient¹s next of kin could not be obtained despite all reasonable attempts. The case is important to public health and every effort has been made to protect the identity of our patient. There is no reason to believe that our patient's next of kin would object to publication.

## Competing interests

The author declares that they have no competing interests.
